# Modeling Superimposed Preeclampsia Using Ang II (Angiotensin II) Infusion in Pregnant Stroke-Prone Spontaneously Hypertensive Rats

**DOI:** 10.1161/HYPERTENSIONAHA.118.10935

**Published:** 2018-05-29

**Authors:** Hannah L. Morgan, Elaine Butler, Shona Ritchie, Florian Herse, Ralf Dechend, Elisabeth Beattie, Martin W. McBride, Delyth Graham

**Affiliations:** 1 From the BHF Glasgow Cardiovascular Research Centre, Institute of Cardiovascular and Medical Sciences, University of Glasgow, United Kingdom (H.L.M., E. Butler, S.R., E. Beattie, M.W.M., D.G.); 2 Experimental and Clinical Research Center, a Joint Cooperation Between the Max-Delbrück Center for Molecular Medicine and the Charité Medical Faculty, Berlin, Germany (F.H., R.D.); 3 HELIOS Clinic Berlin-Buch, Germany (F.H., R.D.).

**Keywords:** angiotensin II, animal model, hypertension, pre-eclampsia, pregnancy

## Abstract

Hypertensive disorders of pregnancy are the second leading cause of maternal deaths worldwide. Superimposed preeclampsia is an increasingly common problem and often associated with impaired placental perfusion. Understanding the underlying mechanisms and developing treatment options are crucial. The pregnant stroke-prone spontaneously hypertensive rat has impaired uteroplacental blood flow and abnormal uterine artery remodeling. We used Ang II (angiotensin II) infusion in pregnant stroke-prone spontaneously hypertensive rats to mimic the increased cardiovascular stress associated with superimposed preeclampsia and examine the impact on the maternal cardiovascular system and fetal development. Continuous infusion of Ang II at 500 or 1000 ng/kg per minute was administered from gestational day 10.5 until term. Radiotelemetry and echocardiography were used to monitor hemodynamic and cardiovascular changes, and urine was collected prepregnancy and throughout gestation. Uterine artery myography assessed uteroplacental vascular function and structure. Fetal measurements were made at gestational day 18.5, and placentas were collected for histological and gene expression analyses. The 1000 ng/kg per minute Ang II treatment significantly increased blood pressure (*P*<0.01), reduced cardiac output (*P*<0.05), and reduced diameter and increased stiffness of the uterine arteries (*P*<0.01) during pregnancy. The albumin:creatinine ratio was increased in both Ang II treatment groups (*P*<0.05; *P*<0.0001). The 1000 ng/kg per minute–treated fetuses were significantly smaller than vehicle treatment (*P*<0.001). Placental expression of Ang II receptors was increased in the junctional zone in 1000 ng/kg per minute Ang II–treated groups (*P*<0.05), with this zone showing depletion of glycogen content and structural abnormalities. Ang II infusion in pregnant stroke-prone spontaneously hypertensive rats mirrors hemodynamic, cardiac, and urinary profiles observed in preeclamptic women, with evidence of impaired fetal growth.

## Abstract 2

Supplemental Digital Content is available in the text.

Hypertensive disorders of pregnancy range from mild gestational hypertension to severe preeclampsia. Prevalence is increasing worldwide as many pregnant women have a higher chance of developing complications because of possessing risk factors prepregnancy, such as advanced maternal age, increased body mass index, obesity, and preexisting hypertension.^1–4^ An increasingly common gestational hypertensive disorder is superimposed preeclampsia, which is the development of preeclampsia superimposed on a background of preexisting chronic hypertension. These conditions are multifactorial, and like human essential hypertension, the causes are not fully understood.^5–7^

Major cardiovascular adaptations are needed to sustain healthy pregnancy; for example, an elevated cardiac output (CO; ≈40% greater than nonpregnant) and increased heart rate (10–20 bpm higher than nonpregnant) usually associated with decreased blood pressure.^8^ This fall in blood pressure is a result of reduced vascular resistance and altered sensitivity to the renin–angiotensin system (RAS), specifically a reduced sensitivity to Ang II (angiotensin II).^8,9^ The main extrarenal source of the RAS is the placenta, which increases expression of all RAS components during pregnancy.^10–13^ However, a reduction in RAS component expression and hypersensitivity to Ang II has been observed in women with gestational hypertensive disorders.^12,14,15^ These disorders are associated with increased blood pressure and systemic vascular dysfunction, which ultimately have a detrimental impact on the offspring.^16^

Ang II infusion is commonly used for generating experimental models of hypertension in wild-type rodents, promoting hypertrophy, fibrosis of the heart, and increased vasoconstriction.^17–20^ Although pregnancy is associated with reduced sensitivity to Ang II,^9^ manipulation of the RAS by increasing Ang II systemically in normotensive rodent pregnancy can mimic preeclamptic phenotypes, such as impairment of vascular remodeling and trophoblast invasion and increased cardiovascular load, which subject the dams to the greater cardiovascular stress.^16,21^ In these models, Ang II causes an increase in blood pressure, placental inflammation, and fetal growth restriction (FGR).^16,21,22^

Pregnant stroke-prone spontaneously hypertensive (SHRSP) rats have constantly elevated blood pressure, compared with the normotensive Wistar Kyoto (WKY) strain, and are hypertensive throughout pregnancy. SHRSP rats also demonstrate aspects of preeclampsia, such as reduced uteroplacental blood flow and impaired uterine artery function in late pregnancy.^23^ These vascular changes have been found to be independent of preexisting hypertension and are not observed in WKY pregnancy. Yet, the SHRSP are still able to maintain pregnancy and produce viable offspring.^23^

In this study, we have used Ang II infusion for the first time in a rat model of chronic hypertension during pregnancy. Our aim was to generate a novel model of superimposed preeclampsia for the investigation of Ang II dysregulation on pregnancy and fetal growth. We hypothesized that Ang II infusion would imitate superimposed preeclamptic phenotypes in pregnant SHRSP. This would include an increased hypertensive insult on the maternal cardiovascular system of the pregnant SHRSP, impacting the maternal cardiovascular system response to pregnancy and the development of the offspring.

## Materials and Methods

A detailed description of methods is available in the online-only Data Supplement. Data supporting these findings are available from the corresponding author on reasonable request.

### Animals

SHRSP and WKY rats, bred and maintained at the University of Glasgow, were housed in controlled 12-hour light/dark conditions with a constant temperature (21°C±3°C) with ad libitum access to water and standard diet (rat and mouse No.1 maintenance diet, Special Diet Services). All animal procedures were approved by the Home Office according to the Animals (Scientific Procedures) Act 1986 (Project License 60/9021). Virgin females were time mated at 12 weeks (±4 days) of age with stud males of the respective strain. Gestational day (GD) 0.5 was confirmed by presence of a copulation plug. Animals were excluded at this stage if pregnancy was not confirmed. Pregnant SHRSP rats were randomly assigned to 1 of 3 treatment groups (0.9% saline vehicle or Ang II at either 500 or 1000 ng/kg per minute); numbers of rats used per treatment are detailed in Table S1 in the online-only Data Supplement. Pregnant WKY rats were used for baseline measurements only and were not assigned to an Ang II treatment group. Dams where blood pressure was being monitored by radiotelemetry were allowed to progress to parturition, and others were euthanized at GD18.5 for tissue collection.

### Ang II Infusion

Primed Alzet 2002 minipumps were implanted at GD10.5 subcutaneously on the right flank of the dam under anesthesia (2% isoflurane in 1.5 L/min oxygen). A constant infusion of either 500 or 1000 ng/kg per minute Ang II (Sigma) was delivered or 0.9% sterile saline as vehicle controls. Doses were chosen based on previous studies conducted in pregnant Sprague Dawley rats.^22^ Minipumps operated at a constant rate of 5.0 µL/h (±0.75 µL/h) for 14 days or until euthanasia.

### Blood Pressure Measurement

A radiotelemetry transmitter was implanted in 10-week-old SHRSP females, as previously described.^24^ Systolic and diastolic blood pressures, heart rate, and activity were continuously monitored prepregnancy and throughout gestation and parturition using the Dataquest system (Data Sciences International).

### Echocardiography and Uterine Artery Doppler

Echocardiography and uterine artery Doppler waveform recordings were performed prepregnancy, at GD6.5, GD14.5, and GD18.5. Rats were anaesthetized (1.5% isoflurane in 1.5 L/min oxygen), and imaging of the heart and uterine artery blood flow was conducted using an Acuson Sequoia c256 ultrasound imager fitted with a 15-MHz linear array transducer. Detailed description of echocardiography and uterine artery Doppler measurements and calculations can be found in Methods in the online-only Data Supplement.

### Biochemical Urinary Analysis

Metabolic cages were used for 24-hour urine collections at GD6.5 and GD14.5 time points. Urine was analyzed for albumin and creatinine concentration using Roche Cobas C311 Analyser and commercially available rodent kits (Roche).

### Uterine Artery Myography

Uterine arteries were dissected in calcium-free physiological salt solution. One- to two-mm sections from the central portion of these arteries were mounted on a Danish Myo Technology multiwire myograph system 620 mol/L in physiological salt solution with Ca^2+^ with 95% O_2_ and 5% CO_2_. These vessels were normalized and checked for viability as previously described.^23^ The contractile dose response to noradrenaline was assessed (1×10^−8^ to 1×10^−5^ mol/L), and relaxation response to carbachol and sodium nitroprusside was assessed (1×10^−9^ to 2×10^−5^ mol/L) after pre-constriction with 2×10^−5^ mol/L noradrenaline. External and internal diameters were determined over a range of physiological pressures (10–120 mm Hg) using a pressure myograph system (Danish Myo Technology) in calcium-free physiological salt solution. Wall thickness, cross-sectional area, wall stress, and wall strain were then calculated (details provided in Methods in the online-only Data Supplement).

### Fetal Weight Distribution

Individual fetal weights at GD18.5 were recorded and pooled for each treatment group. Histograms of these weights were created using the relative percentage frequencies for each weight, and a nonlinear regression was then performed using a Gaussian distribution. The relative frequency distributions were used to determine which weight represented the lowest fifth percent of vehicle fetal weights (fifth centile).

### Placental Gene Expression

RNA was extracted from mesometrial, junctional, and labyrinth zones of the placenta using the miRNeasy mini kit (Qiagen) according to manufacturer’s instructions. cDNA templates were produced, and Taqman quantitative polymerase chain reaction was performed for components of the RAS (Ang II receptor type-1 [Agtr1], Ang II receptor type-2 [Agtr2], and angiotensin-converting enzyme 2 [Ace2]), markers of oxidative stress (hypoxia-inducible factor 1-alpha [Hif1α] and superoxide dismutase 1 [Sod1]), and inflammation (C-C chemokine receptor type 11 [Ccrl1] and C-C chemokine ligand 2 [Ccl2]). See Table S2 for primer sequences.

### Histology

Periodic acid Schiff stain was used to assess morphological differences of the maternal kidney and placenta.^23^ Placental glycogen cell content was determined by the percentage of positive magenta staining in the junctional zone analyzed using Image J software. Placentas were also scored for junctional disorganization (see online-only Data Supplement for scoring details).

### Statistical Analysis

Coding was used to blind observers to the treatment groups during data acquisition/analysis and decoded before statistical analysis. All data are presented as mean±SEM. Telemetry data are presented as 12-hour averages and were analyzed using area under the curve for pre– and post–Ang II infusion, compared using 1-way ANOVA with Tukey post hoc tests. Regression analysis was used to find the rate of change where longitudinal measurements were made from the same dams over gestation; these gradients were then compared with a null hypothesis mean of 0 using a 1-sample *t* test to determine gestational dependent change. All other comparisons of the 3 treatment groups were analyzed using 1-way ANOVA with a Tukey post hoc test unless otherwise stated. Gene expression analysis was conducted using the delta cycle threshold (dCT) values. A *P*<0.05 was deemed to represent a statistically significant result.

## Results

### Blood Pressure Profile During Ang II–Infused Pregnancy

**Figure 1.**
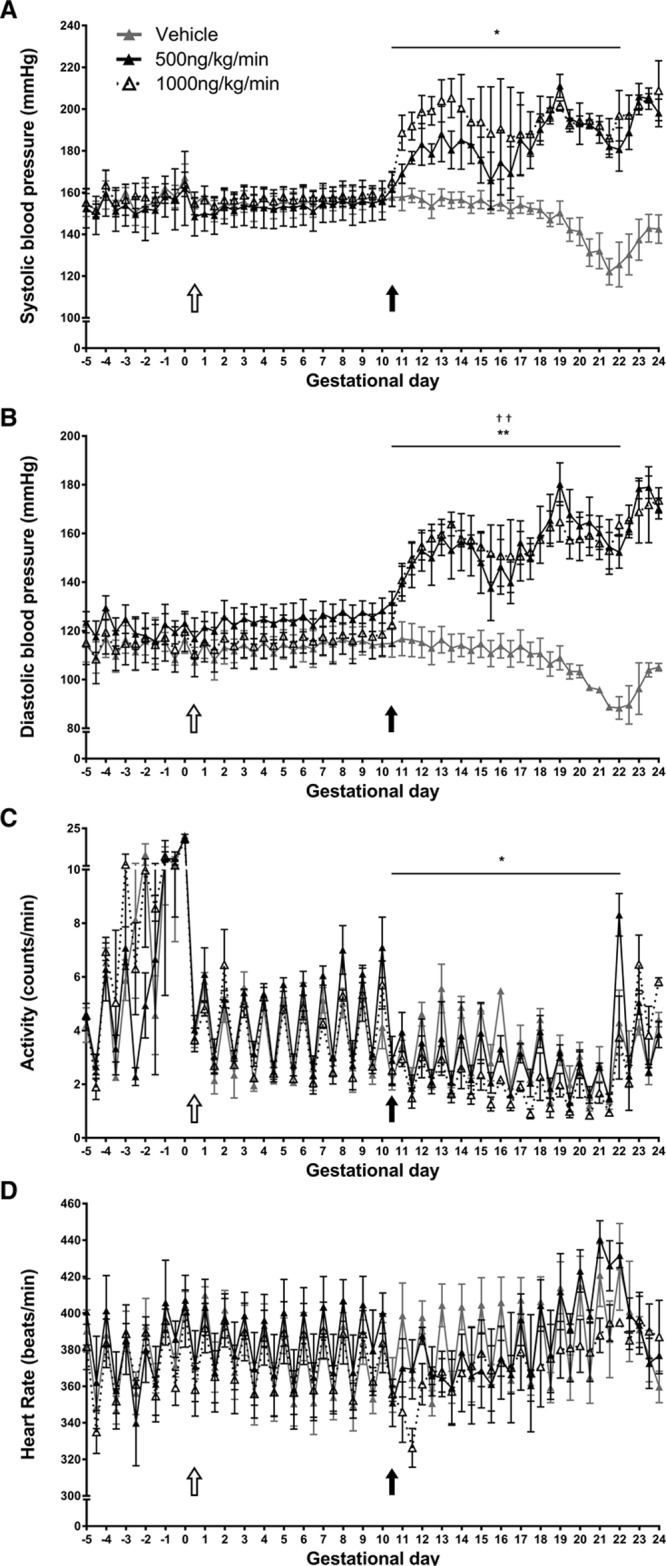
Radiotelemetry data show systolic and diastolic blood pressures (**A**–**B**), activity (**C**), and heart rate (**D**). Negative gestational days (GDs) indicate prepregnancy; GD0.5 to GD10.5 indicates pregnancy before Ang II (angiotensin II) infusion. Ang II or vehicle infusion began on GD10.5 and was sustained until parturition (approximately GD22). Open arrows indicate conception; solid black arrows indicate minipump implantation. Area under the curve was compared using 1-way ANOVA for pre–Ang II infusion (GD0.5–GD10.5) and post–Ang II infusion (GD10.5–GD21.5); n=3 to 4; *P<0.05, **P<0.01 for 1000 ng/kg per minute vs vehicle; ††*P*<0.01 for 500 ng/kg per minute vs vehicle.

Ang II infusion from GD10.5 caused significant increases in systolic and diastolic blood pressures (Figure 1A and 1B). The rise in systolic pressure reached significance in the 1000 ng/kg per minute Ang II treatment group (192±2.1 versus 144±2.3 mm Hg; *P*<0.05), and diastolic pressure was significantly increased in both the 500 ng/kg per minute Ang II and the 1000 ng/kg per minute Ang II treatment groups (157±2.3 and 158±2.2 versus 105±1.7 mm Hg; *P*<0.01, respectively) compared with vehicle. Dams treated with 1000 ng/kg per minute Ang II were significantly less active than the vehicle group during the nocturnal (awake) periods (2.4±0.3 versus 3.1±0.2 counts/min; *P*<0.05; Figure 1C). Mean heart rate was not significantly affected by Ang II treatment (Figure 1D); however, the 1000 ng/kg per minute Ang II treatment group lost the typical amplitude in diurnal rhythm.

### Cardiac and Vascular Parameters

**Figure 2.**
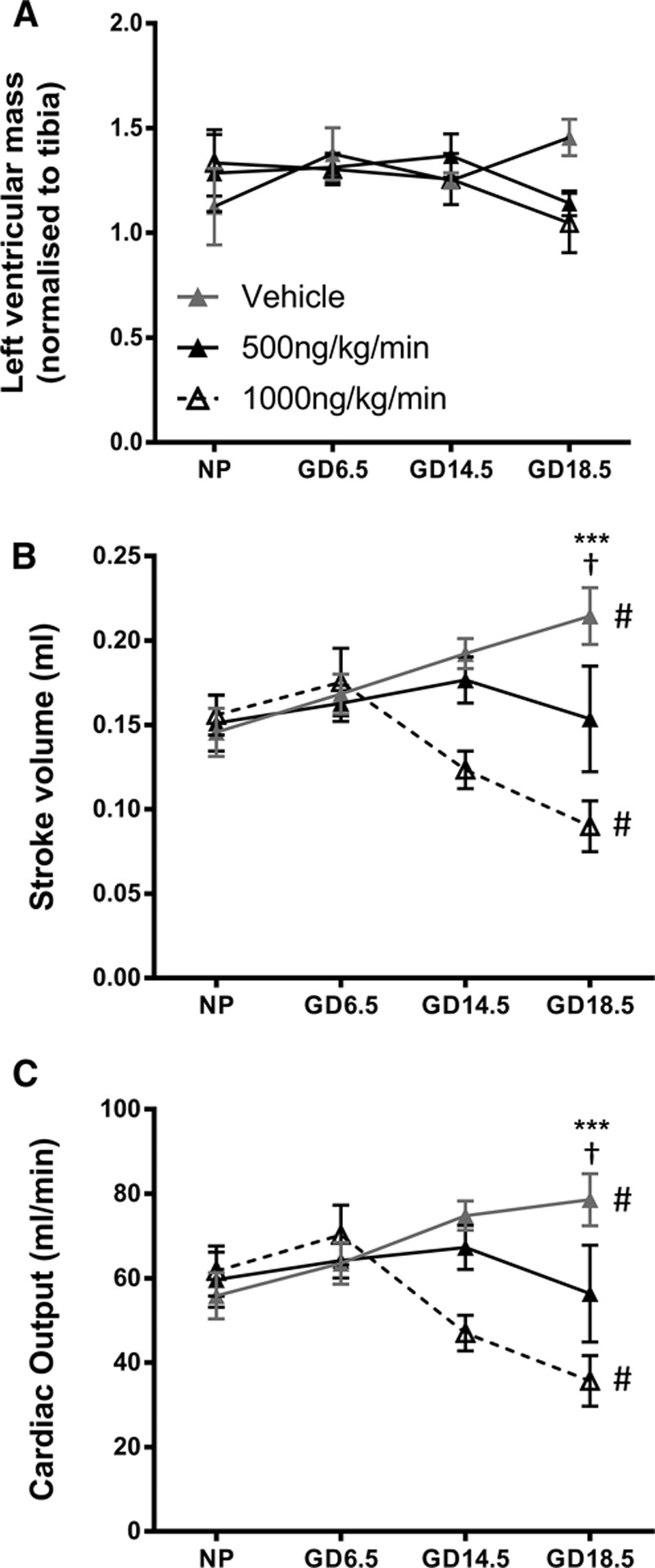
Echocardiography was conducted prepregnancy (NP), pre–Ang II (angiotensin II; gestational day [GD] 6.5), and post–Ang II infusion (GD14.5 and GD18.5). **A**, Left ventricular mass was normalized to tibia length and was unchanged over pregnancy regardless of Ang II treatment. Pregnancy increased stroke volume (**B**) and cardiac output (**C**) in the vehicle group (n=5) while had no effect on dams treated with 500 ng/kg per minute Ang II (n=4). Stroke volume and cardiac output were significantly reduced in 1000 ng/kg per minute Ang II–treated dams (n=6). One sample *t* tests compared the gradients pre–Ang II infusion (NP to GD6.5; ns) and post–Ang II infusion (GD6.5–GD18.5; #*P*<0.05). One-way ANOVA compared the gradient change between treatment groups post–Ang II infusion (***P<0.001 vehicle vs 1000 ng/kg per minute; †*P*<0.05 500 vs 1000 ng/kg per minute).

No significant differences were observed in left ventricular mass, stroke volume, and CO at GD6.5 (pre–Ang II treatment; Figure 2). Left ventricular mass was not significantly altered by Ang II treatment (Figure 2A). Stroke volume in vehicle-treated SHRSP showed a significant increase post–Ang II infusion (168±12 µL GD6.5 versus 214±17 µL GD18.5; *P*<0.05; Figure 2B). This change was not observed in the 500 ng/kg per minute Ang II treatment group and was significantly reduced in the 1000 ng/kg per minute Ang II treatment group (182±17 versus 90±15 µL; *P*<0.05; Figure 2B). Comparison of the rate of change in stroke volume showed that the 1000 ng/kg per minute Ang II group had a significantly lower stroke volume compared with vehicle (*P*<0.001) and 500 ng/kg per minute Ang II treatment group (*P*<0.05). These same trends were observed in the CO response post–Ang II infusion with vehicle-treated SHRSP demonstrating a significant increase in CO (63±5 mL/min GD6.5 versus 79±6 mL/min GD18.5; *P*<0.05; Figure 2C). No change was observed in the 500 ng/kg per minute Ang II treatment group. The 1000 ng/kg per minute Ang II treatment group demonstrated a significant reduction in CO (73±6 mL/min GD6.5 versus 36±6 mL/min GD18.5; *P*<0.05; Figure 2C). Comparison of the rate of change in CO showed that 1000 ng/kg per minute had a significantly reduced level compared with vehicle (*P*<0.001) and 500 ng/kg per minute Ang II treatment group (*P*<0.05). Representative images of echocardiography traces are shown in Figure S1.

The uterine artery Doppler showed no significant differences in the systolic/diastolic ratio or resistance index between the 3 treatment groups at each gestational age (Figure S2).

### Evidence of Proteinuria and Abnormal Kidney Morphology

**Figure 3.**
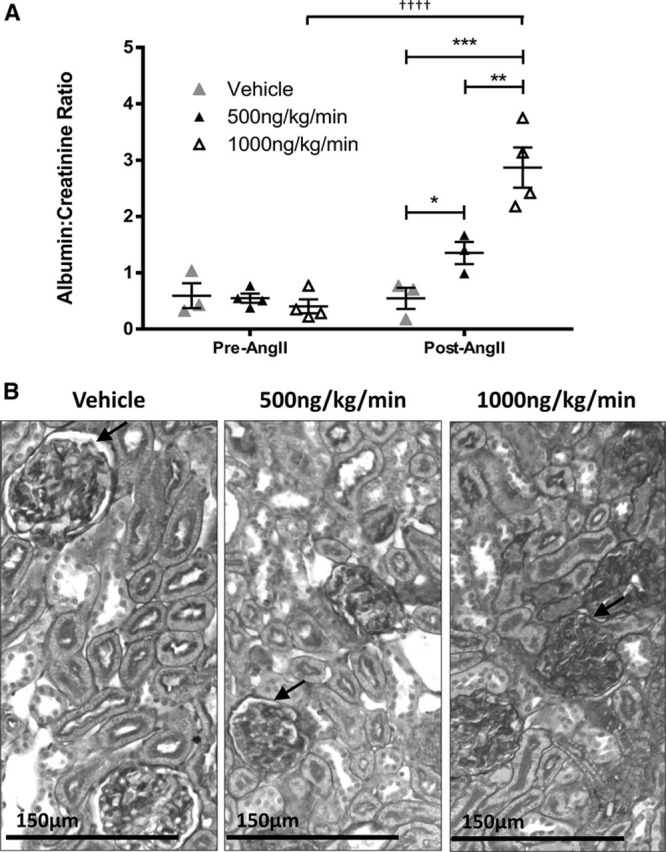
**A**, Urinary albumin:creatinine ratios (ACR) were determined from 24-h urine samples collected pre–Ang II (angiotensin II; gestational day [GD] 6.5) and post–Ang II infusion (GD14.5). Ang II infusion significantly increased ACR at both the 500 ng/kg per minute (n=3) and 1000 ng/kg per minute (n=4) doses. The vehicle-treated group (n=3) demonstrated no pregnancy-dependent change. Two-way ANOVA compared changes between pre–Ang II and post–Ang II infusion; ††††*P*<0.0001 and between treatment groups; *P<0.05, ***P<0.001, ****P<0.0001. **B**, Representative images of periodic acid Schiff–stained kidney sections from GD18.5 vehicle, 500 ng/kg per minute Ang II, and 1000 ng/kg per minute Ang II–treated dams. Arrows indicate Bowman space that appears reduced between the visceral and parietal layers as Ang II treatment increases.

Both high- and low-dose Ang II groups had significantly increased albumin:creatinine ratio compared with vehicle control post–Ang II treatment (1.4±0.2 500 ng/kg per minute and 3.0±0.3 1000 ng/kg per minute versus 0.5±0.2 vehicle; *P*<0.05; *P*<0.0001). Dams treated with 1000 ng/kg per minute Ang II demonstrated a significantly greater albumin:creatinine ratio than 500 ng/kg per minute Ang II treatment group (*P*<0.001) and was the only treatment group to demonstrate a significantly increased ratio from pre–Ang II treatment (0.4±0.1 versus 3.0±0.3; *P*<0.0001; Figure 3A).

The kidneys of Ang II–treated dams demonstrated morphological differences (Figure 3B). Representative images show the typical SHRSP glomeruli structure with clear cell definition within the glomeruli capillaries and visible space between the visceral and parietal layers of the Bowman capsule. There is evidence of Bowman capsule defects in Ang II–treated SHRSP kidneys, with a reduction in the space observed between the visceral and parietal layers.

### Uterine Artery Function and Structure

**Figure 4.**
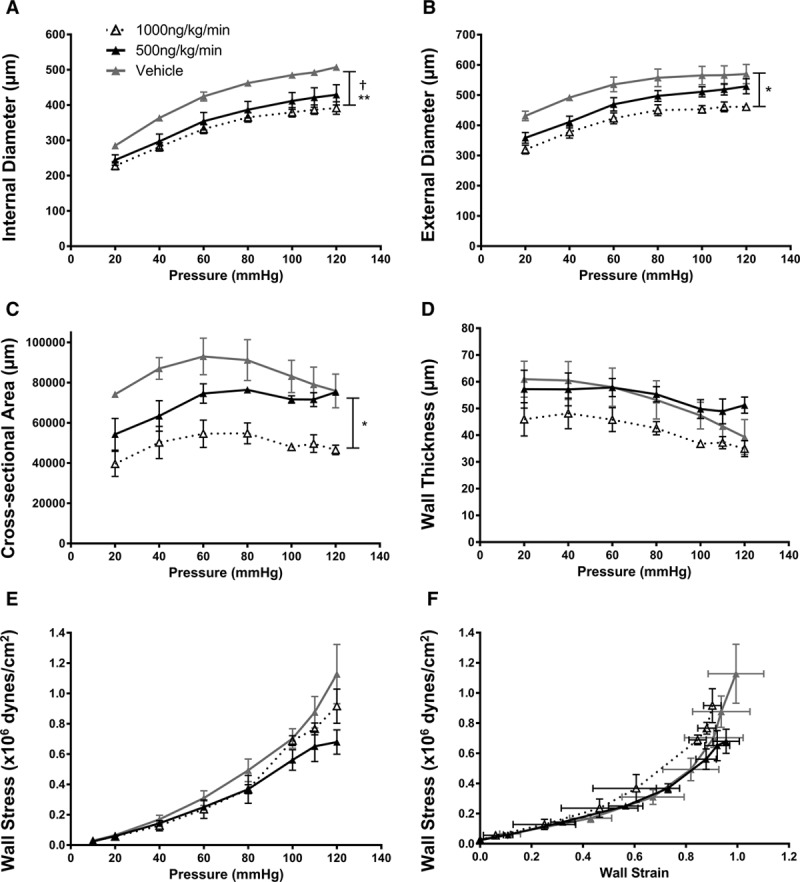
Pressure myography assessment of the wall properties demonstrated that uterine arteries from 1000 ng/kg per min Ang II–treated dams had a significantly reduced diameter (both internal and external; **A** and **B**) and cross-sectional area (**C**), with no changes in wall thickness (**D**). Ang II (angiotensin II) treatment had no significant impact in wall stress (**E**); however, the stress–strain relationship was significantly reduced in both Ang II treatment groups (**F**). Area under the curve compared using 1-way ANOVA; n=3 to 6; *P<0.05, **P<0.01 for 1000 ng/kg per minute vs vehicle; **P<0.01 for 500 ng/kg per minute vs vehicle.

The vascular reactivity of GD18.5 uterine arteries was not significantly altered by Ang II treatment (Figure S3). The uterine arteries from 1000 ng/kg per minute Ang II–treated dams had a significantly reduced external and internal diameter compared with vehicle (2866±101 versus 3535±164 area under the curve; *P*<0.05 and 2271±88 versus 2890±34 area under the curve; *P*<0.01, respectively; Figure 4A and 4B). The cross-sectional area of the vessels from this group was also significantly reduced (3.4×10^5^±2×10^5^ versus 5.6×10^5^±5×10^5^ area under the curve; *P*<0.05; Figure 4C); however, there was no significant differences in wall thickness (Figure 4D) or wall stress (Figure 4E) across the range of physiological pressures. Histopathology revealed no differences in wall cross-sectional area (Figure S4). The vessel stress/strain relationship showed a significant decrease in both Ang II treatment groups (Figure 4F), with the curves shifting to the left, indicating increased vascular stiffness.

### Fetal Impact

**Figure 5.**
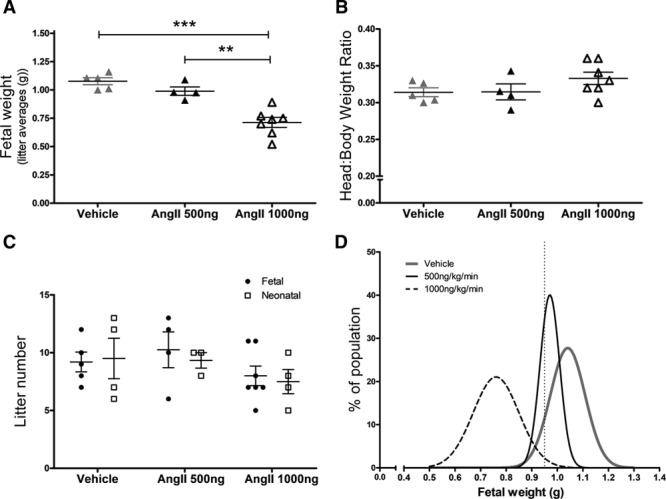
Fetal measures were made at gestational day (GD) 18.5. **A**, Fetal weights were significantly reduced with 1000 ng/kg per minute Ang II (angiotensin II) treatment compared with vehicle and 500 ng/kg per minute. **B**, There was no significant change in head:body weight ratio or (**C**) the number of offspring per litter at the fetal (GD18.5) or neonatal (day 2) stage. Data are presented as litter averages for each dam. **P<0.01, ***P<0.001; compared using 1-way ANOVA. **D**, The fetal growth distributions of vehicle stroke-prone spontaneously hypertensive (SHRSP; n=37) and both Ang II treatment groups (n=42, 500 ng/kg per minute and n=46, 1000 ng/kg per minute) indicate fetuses that would be clinically classed as growth restricted; vertical dotted line represents the fifth centile for SHRSP fetal weights. The 47.8% of 500 ng/kg per minute Ang II–treated fetuses and 95.7% of 1000 ng/kg per minute Ang II–treated fetuses fall below this threshold.

There were no significant differences between untreated WKY and SHRSP fetal weights at GD18.5 (Figure S5). Ang II treatment significantly reduced GD18.5 average fetal weights in the 1000 ng/kg per minute Ang II treatment group compared with both vehicle and 500 ng/kg per minute Ang II treatment groups (0.71±0.04g versus 1.08±0.03g and 0.99±0.04g; *P*<0.0001 and *P*<0.01, respectively; Figure 5A). There were no changes in the head:body weight ratio or in the number of pups per litter at the fetal (GD18.5) or at neonatal (day 2) stage (Figure 5B and 5C). Fetal weight distributions show that 47.8% of 500 ng/kg per minute Ang II fetuses and 95.7% of 1000 ng/kg per minute Ang II fetuses fell below the fifth centile for fetal weight in vehicle SHRSP (Figure 5D).

### Placental Gene Expression and Morphology

**Figure 6.**
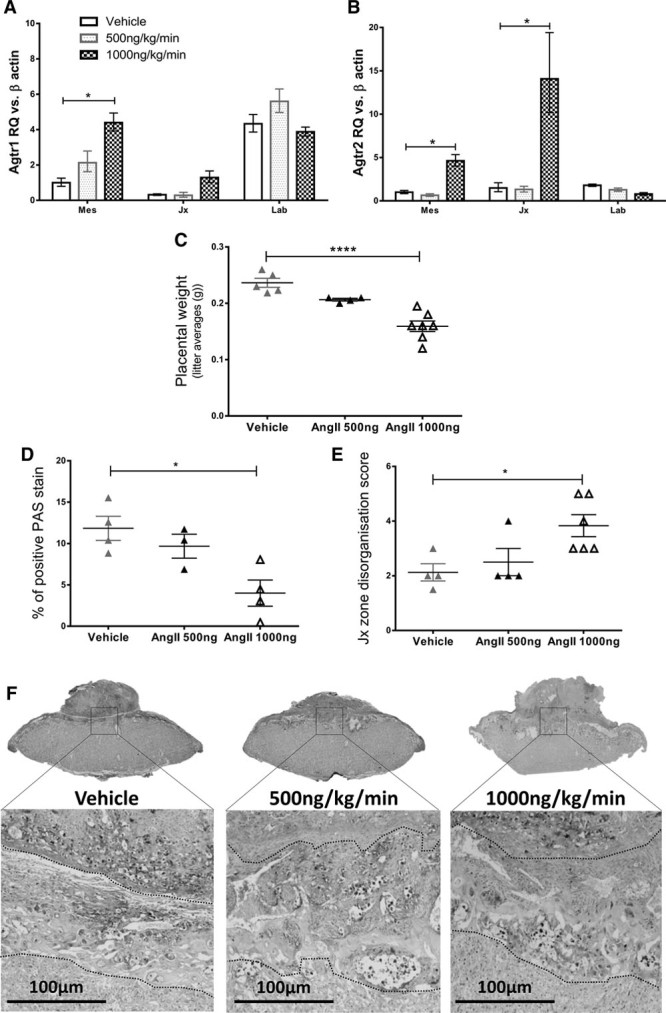
Gene expressions were investigated in the different layers of the placenta **A** and **B**, Ang II (angiotensin II) receptor type-1 and type-2 expression was increased in the mesometrial triangle from 1000 ng/kg per minute Ang II treatment group. Data are presented as relative quantity (RQ) compared with stable housekeeper (β-actin). Delta cycle threshold values analyzed using 1-way ANOVA; *P<0.05. **C**, Placental weights at gestational day (GD) 18.5 were significantly reduced in the 1000 ng/kg per minute Ang II treatment group. **D**, Periodic acid Schiff staining was significantly reduced in placenta exposed to 1000 ng/kg per minute Ang II treatment. **E**, Junctional zone disorganization was scored by a blinded operator (1=organized typical structure, with ordered distribution of cell types; 5=no order, many large vacuous regions with sparse and disorganized distribution of cell types). **C**–**E**, Assessed using 1-way ANOVA with Tukey post hoc test; *P<0.05, ****P<0.0001. **F**, Representative images of whole placentas (merged images) and the junctional zone at ×20 magnification are shown with the junctional zone outlined by dashed lines. Jx indicates junctional zone; laboratory, labyrinth zone (placental tissues); and Mes, mesometrial triangle (maternal tissue including decidua).

The gene expressions of Agtr1 and Agtr2 were significantly increased in the mesometrial triangle of 1000 ng/kg per minute Ang II–treated placentas compared with vehicle (Agtr1: 11.70±0.3 versus 13.84±0.6 dCT; *P*<0.05 and Agtr2: 13.16±0.4 versus 15.37±0.5 dCT; *P*<0.05; Figure 6A and 6B). Agtr2 was also significantly increased in the junctional zone of placenta from 1000 ng/kg per minute Ang II–treated dams compared with vehicle (11.56±0.8 versus 14.80±0.9 dCT; *P*<0.05; Figure 6B). No significant differences were observed in other placental layers. There were no expression changes in Ace2, Sod1, Hif1α, Ccrl1, or Ccl2 in the different layers (Figure S6).

Placental weights were significantly reduced by 1000 ng/kg per minute Ang II treatment compared with vehicle (0.16±0.009 versus 0.24±0.008 g; *P*<0.0001; Figure 6C). This reduction in placental weight was accompanied with a loss of glycogen content. Periodic acid Schiff staining showed significantly less glycogen positive cells in the junctional zone of the 1000 ng/kg per minute Ang II group compared with vehicle (4%±1.6% versus 13%±1.5%; *P*<0.05; Figure 6D). The junctional zone of the placenta also exhibited a significant increase in disorganization score in the 1000 ng/kg per minute Ang II treatment group (4±0.5 versus 2±0.3; *P*<0.05; Figure 6E). Representative images of the whole-rodent placenta and junctional zone at higher magnification are shown in Figure 6F.

## Discussion

This study has shown that administration of Ang II during hypertensive pregnancy can significantly impact maternal cardiovascular physiology and the growth of the fetus. We have demonstrated that infusion of high dose Ang II from midgestation in SHRSP pregnancy increases maternal blood pressure, with reduced stroke volume and thus reduced CO. The 500 ng/kg per minute Ang II dose caused a significant increase in blood pressure and proteinuria and lacked the significant increase in CO observed in vehicle-treated SHRSP rats. However, we did not find any significant reduction in fetal weight in this group compared with vehicle. The 1000 ng/kg per minute Ang II dose demonstrated the greatest increase in blood pressure and a severe impairment of CO. This impact on the maternal cardiovascular system was observed in parallel with the development of proteinuria and reduced fetal weight, suggesting that this high Ang II dose is analogous to more extreme human preeclamptic characteristics.^25,26^ Human preeclampsia has been associated with the development of FGR, and offspring from preeclampsia pregnancies have been found to be more at risk of cardiovascular disease.^16,27^ Dysregulation of the RAS in utero has been associated with the development of hypertension in later life.^16,27,28^ Xue et al^16^ demonstrated that offspring exposed to low pressor Ang II in utero were hypersensitive to Ang II–induced hypertension as adults. These offspring were more likely to develop cardiovascular disease because of differences in developmental programming compared with untreated counterparts.^16^

Ang II treatment caused uterine artery diameter and cross-sectional area reductions, suggesting that the pregnancy-specific outward hypertrophic remodeling of these arteries does not occur to the same extent as that observed in untreated animals.^23^ This is in line with the human situation, where a major defect in myometrial spiral artery remodeling can be observed in preeclampsia and FGR.^29^ We have previously shown that SHRSP demonstrate pregnancy-dependent uterine artery remodeling compared with nonpregnant SHRSP; however, this remodeling is impaired when compared with WKY rats.^23^ It is unclear whether the SHRSP Ang II infusion model is preventing the outward hypertrophic remodeling or reversing any pregnancy-dependant changes. Measurements of vessel diameter at GD10.5 before Ang II infusion would be needed to answer this. Previously, we have demonstrated that SHRSP have an impaired uteroplacental blood flow and impaired vasorelaxation responses, so it is possible that the observed lack of functional changes of the uterine arteries in the present study occur because of preexisting impairment that cannot be worsened by Ang II infusion.^23^

Circulating Ang II levels in preeclampsia are similar to those found in normotensive pregnancies^9^; however, the receptor sensitivity and downstream signaling processes are increased.^9,14,30^ We demonstrated that preeclampsia-like symptoms can be developed midpregnancy in an animal model using Ang II infusion from GD10.5. This is in line with human pregnancies where preeclampsia symptoms do not arise until after 20 weeks gestation.^31,32^ Local tissue Ang II production plays a crucial role in RAS maintenance during pregnancy, and RAS components have been found in both rodent and human placenta.^11,33–35^ However, any local control of Ang II production by the placenta may be masked by exogenous administration in our model. Placental specific increases of Ang II, achieved using transgenic crosses, have shown similar preeclampsia phenotypes to those observed in our SHRSP Ang II infusion model.^21,34,36^ We found evidence of increased expressions of *Agtr1* and *Agtr2* genes in the mesometrial triangle from 1000 ng/kg per minute Ang II–treated dams. In human decidua, an increase in Agtr1 has been described in preeclampsia.^37^ Furthermore, an increase in Agtr1 activation via Ang II has been found to reduce the placental transport of system A amino acids.^38^ Vaswani et al^10,11^ found a gestational-dependent increase in the gene expression of components of the RAS (eg, Ace2 and Agtr1a) in rodent placenta. This study suggests that the placental RAS is dynamic throughout pregnancy and is influenced by Ang II changes. Our SHRSP Ang II infusion model alters the RAS once the rodent placental cell lineages are established during the formation of the chorioallantoic placenta. However, this does not result in decreased numbers of fetuses per litter, suggesting Ang II infusion may be causing placental dysfunction rather than a failure of placental development. Other placental defects demonstrated by Ang II infusion in SHRSP were a reduction in placental weight, as well as depletion and disorganization of the junctional zone. Because placental dysfunction has been well established in its involvement with FGR in humans,^39–41^ it is possible that these placental deficiencies have a significant impact on sustaining the fetus and thus promote the development of FGR in this model.

In this study, we investigated the in vivo cardiovascular changes across gestation in the SHRSP. CO has been found to increase by up to 40% to accommodate the need for increased organ perfusion during human pregnancy.^8^ Pregnancy is suggested to be a stress test for the cardiovascular system, with preeclampsia resulting because of a failure to mount an appropriate response. Severe preeclampsia is associated with cardiovascular impairment; specifically the increase in CO is often reduced or absent.^26^ This has been associated with poor placental perfusion and presence of FGR.^26,42,43^ It is still not known whether the cardiovascular impairment occurs before the placental dysfunction or whether it is the dysfunction that causes systemic cardiovascular stress that in turn makes the situation worse. However, there is increasing evidence that the maternal cardiovascular system’s response to pregnancy is a major contributor to the development of gestational hypertensive disorders.^44^ Vehicle-treated SHRSP demonstrated normal CO changes during pregnancy (increasing by 53% from nonpregnant) whereas SHRSP treated with 1000 ng/kg per minute Ang II demonstrated a 40% reduction in CO over pregnancy.

Two Ang II doses (low 500 ng/kg per minute and high 1000 ng/kg per minute) were investigated using the minipump infusion model in this study. Women with gestational hypertensive complications have varying degrees of severity, and previous studies in normotensive rodents have used doses ranging from 500 to 1000 ng/kg per minute.^16,22,30^ The method described in the current study creates a robust, easily reproducible model of preeclampsia with potential to vary the cardiovascular insult by modifying the Ang II dose administered. Pregnant women are more resistant to the effects of Ang II compared with nonpregnant women; yet those that later develop pregnancy-induced hypertension have been found to have increased sensitivity.^9^ This model could, therefore, be adapted to provide information about an extreme condition (1000 ng/kg per minute) versus mild (500 ng/kg/minute). Our model differs from previous Ang II infusion models mimicking preeclampsia symptoms because SHRSP rats are hypertensive before pregnancy. We suggest that this model represents clinically observed superimposed preeclampsia, where the onset of preeclampsia occurs on a background of preexisting hypertension and is a condition that is relatively less well studied compared with other pregnancy-induced hypertensive conditions.^45^ This model is novel because it imposes a pregnancy-dependent increase in hypertension on a model with chronically elevated blood pressure. Considering the increasing incidence of women with cardiovascular risk factors becoming pregnant in Western society, it is plausible that these women possess a mild hypertensive condition which remains undiagnosed because screening for hypertension is not regularly conducted in women <50 years.^6,46^ However, it has been suggested that women who experience preeclampsia may have a predisposition to cardiovascular and metabolic diseases, with pregnancy highlighting these women at risk.^7^

## Perspectives

Ang II infusion can be used to create a superimposed preeclampsia-like phenotype in pregnant SHRSP rats. This model could be adapted to vary the cardiovascular stress imposed on the mother during pregnancy and will be a useful tool for investigating the impact of gestational hypertension on maternal cardiovascular pathophysiology, as well as the effect this has on offspring later in life. This model will aid investigations into preeclampsia when studied in parallel with other animal models and will be useful in determining underlying mechanisms of gestational hypertension and in the assessment of novel therapeutic strategies.

## Sources of Funding

This work was supported by funding from the Medical Research Council (1521437) and the British Heart Foundation Centre of Excellence award (RE/13/5/30177). The Deutsche Forschungsgemeinschaft supported Dr Herse (HE 6249/5-1).

## Disclosures

None.

## Supplementary Material

## Novelty and Significance

### What Is New?

- The pregnant stroke-prone spontaneously hypertensive rat infused with angiotensin II from midgestation provides a novel model of superimposed preeclampsia.

### What Is Relevant?

- Superimposed preeclampsia is becoming increasingly common because of increased prevalence of mothers with risk factors for hypertension prepregnancy.
- Development of relevant animal models is crucial for the investigation of disease mechanisms and development of novel treatment options.

### Summary

Ang II (angiotensin II) infusion in pregnant stroke-prone spontaneously hypertensive rats produces preeclamptic-like phenotypes that mirror hemodynamic, cardiovascular, and renal pathologies observed in preeclamptic women. This provides an easily reproducible rodent model to investigate underlying mechanisms in the development of preeclamptic like conditions.
