# Supplementary material for: Modeling Superimposed Preeclampsia Using Ang II (Angiotensin II) Infusion in Pregnant Stroke-Prone Spontaneously Hypertensive Rats
Source: Hypertension. 2018 May 29;72(1):208–18. doi: 10.1161/HYPERTENSIONAHA.118.10935 (PMC6012051; doi:10.1161/HYPERTENSIONAHA.118.10935)
Supplement: Supplementary file 1 [file hyp-72-208-s001.docx]

# ONLINE SUPPLEMENT

# MODELLING SUPERIMPOSED PREECLAMPSIA USING ANGIOTENSIN II INFUSION IN PREGNANT STROKE PRONE SPONTANEOUSLY HYPERTENSIVE RATS

Hannah Louise Morgan^1^, Elaine Butler^1^, Shona Ritchie^1^, Florian Herse^2^, Ralf Dechend^2^, Elisabeth Beattie^1^, Martin W McBride^1^, Delyth Graham^1^

^1^ BHF Glasgow Cardiovascular Research Centre, Institute of Cardiovascular and Medical Sciences, University of Glasgow, Scotland, UK

^2^ Experimental and Clinical Research Center, a joint cooperation between the Max-Delbrück Center for Molecular Medicine and the Charité Medical Faculty, Berlin, and HELIOS Clinic, Berlin-Buch, Germany

Corresponding Author:

Miss Hannah Louise Morgan

BHF Glasgow Cardiovascular Research Centre, 126 University Avenue, University of Glasgow, Glasgow, G12 8TA

Tel: 0141 3305074

Email: h.morgan.1@research.gla.ac.uk

**Supplementary Methods**

**Echocardiography calculations**

Anterior and posterior wall thickness and end diastolic and systolic diameters of the heart were measured from 3 consecutive cardiac cycles to determine left ventricular mass, stroke volume and cardiac output. Analysis of the echocardiograph images was conducted using Image J software. Distance between waveform peaks and troughs were measured to determine anterior wall thickness, posterior wall thickness; in systole and diastole, end diastolic diameter and end systolic diameter. These measures were used in the following formulae to calculate the left ventricular mass (LVM), stroke volume (SV) and cardiac output (CO). Formulae:

- *Left ventricular mass (g) = ((0.8 × ASEcube)+0.6)⁄1000*
- *Stoke volume (ml) = EDDV⁄ESSV*
- *Cardiac output (ml/min) = SV ×HR*
- *HR (beats/min)*

Calculations were based on recommendations from the American Society of Echocardiography (ASE)^1^.

**Pulse-wave Doppler calculations**

Uterine artery Doppler systolic (S) and diastolic (D) peaks were recorded for 6 consecutive cardiac cycles. Peak systolic velocity (PSV) and end diastolic velocity (EDV) of the uterine arteries were measured from the 6 consecutive cardiac cycles, using Image J, and used to calculate resistance index (RI) (RI = [PSV-EDV]/PSV) and S/D ratio (PSV/EDV).

**Uterine Artery Pressure Myography Calculations**

The external (D_E_) and internal (D_I_) diameter were measured at each pressure. Wall thickness (µm) = (D_E_ - D­_I_)/2; cross-sectional area (µm^2^) = π/4 x (D_E_^2^ – D_I_^2^); circumferential wall strain = (D_I_ - D_I@10mmHg_)/ D_I@10mmHg_ and wall stress (dynes/cm^2^) = 1332 x pressure x D_I_ / (2 x wall thickness), where 1mmHg = 1332 dynes/cm^2^.

**Histological Junctional Zone Scoring**

The whole junctional zone of the placenta was examined and scored from 1 to 5 by a blinded observer, with 1 = organised typical structure, with ordered distribution of cell types; 5 = no order and many large vacuous regions with sparse and disorganised distribution of cell types.

Supplementary reference:

1. Park SH, Shub C, Nobrega TP, Bailey KR, Seward JB. Two-dimensional echocardiographic calculation of left ventricular mass as recommended by the American Society of Echocardiography: correlation with autopsy and M-mode echocardiography. *J Am Soc Echocardiogr.* 1996;9(2):119-128.

**Supplementary Tables and Figures**

**Supplemental Table S1:** Number of dams in each treatment group used for different experimental investigations.

| **AngII Treatment**  **Group**  **Experimental condition** | **Vehicle** | **500ng/kg/min** | **1000ng/kg/min** |
| --- | --- | --- | --- |
| **Radiotelemetry** (Study 1) | 3 | 3 | 4 |
| **Fetal and placental assessment** (Study 2) | 5 | 4 | 7 |
| **Biochemical Urinary Analysis** (Study 2) | 3 | 3 | 4 |
| **Echocardiography / Doppler ultrasound** (Study 2) | 5 | 4 | 6 |
| **Vessel Myography** (Study 2) | 4 | 4 | 6 |

WKY untreated dams used as reference for fetal weights n=29.

**Supplemental Table S2**: Details of Taqman qPCR probe used for gene expression analysis of the placental layers. All purchased from ThermoFisher Life Scientific.

Supplemental Figure S1: Representative echocardiography of the three treatment groups at GD18.5. Each show 3 consecutive cardiac cycles and measurements were made of the anterior and posterior wall thickness and end diameter in systole and diastole.

(C)

(B)

(A)

Supplemental Figure S2: Uterine artery Doppler imaging was used to determine the systolic/diastolic ratio and resistance index at pre-pregnancy (NP), pre-AngII infusion (GD6), post AngII infusion (GD14 and GD18) time points. (A) Representative NP and GD18.5 Doppler traces for each treatment group are presented. (B) There were no significant differences in the systolic/diastolic ratio or resistance index with either AngII treatment compared to vehicle. Vehicle group (n=5), 500ng/kg/min AngII group (n=4) and 1000ng/kg/min AngII group (n=6). Assessed using two-way ANOVA with Tukey post-hoc test.

Supplemental Figure S3: GD18.5 uterine artery wire myography showed no significant changes in contractile response to noradrenaline (A) or relaxation responses to carbachol (B) or sodium nitroprusside (SNP) (C) with AngII treatment. Analysed using area under the curve compared by one-way ANOVA; n=3-6.

Supplemental Figure S4: Representative images of the uterine artery after fixing at 120mmHg and examining cross-sectional area using elastin Verhoeff-van Gieson stain. This analysis revealed no differences in total cross-sectional area between treatment groups.

Supplemental Figure S5: (A) The number of pups born to each litter was recorded for 29 Wistar Kyoto (WKY) pregnancies and 23 SHRSP pregnancies. (B) Fetal weights measured at gestational day 18.5 in the normotensive WKY and hypertensive SHRSP rats. Each point represents the average fetal weight of one litter. WKY n=25 dams, SHRSP n=20 dams; compared using students t-test. (C) The percentage of fetal weight distributions for WKY and SHRSP indicates normal distributions of similar weights in both strains. WKY n= 228 individual fetal weights, SHRSP n= 205 individual fetal weights.

Supplemental Figure S6: qPCR gene expression of (A) Ace2, (B) Sod1, (C) Hif1α, (D) Ccl2 and (E) CcrL1 expressed as relative quantity (RQ) compared to β-actin. There wereno expression differences due to AngII treatment in different layers of the placenta; Mes = mesometrial triangle (maternal tissue including decidua), Jx = junctional zone, Lab = labyrinth zone (placental tissues); n=4, statistical analysis on dCT values compared using one-way ANOVA.
